# Association Between Adjuvant Therapy and Survival in Resected Pancreatic Ductal Adenocarcinoma After Different Types and Durations of Neoadjuvant Therapy

**DOI:** 10.1245/s10434-025-17439-x

**Published:** 2025-05-29

**Authors:** Paul C. M. Andel, Brady A. Campbell, Joseph R. Habib, I. Quintus Molenaar, Kelly J. Lafaro, William R. Burns, Lois A. Daamen, John L. Cameron, Christopher L. Wolfgang, Richard A. Burkhart, Jin He, Ammar A. Javed

**Affiliations:** 1 Department of Surgery, The NYU Grossman School of Medicine and NYU Langone Health, New York, NY USA; 2 Department of Surgery, Regional Academic Cancer Center Utrecht, UMC Utrecht Cancer Center and St Antonius Hospital Nieuwegein, Utrecht, The Netherlands; 3 Department of Surgery, Johns Hopkins University School of Medicine, Baltimore, MD USA; 4 Imaging Division, UMC Utrecht Cancer Center, Utrecht, The Netherlands; 5 Department of Surgery, Amsterdam UMC, Location University of Amsterdam, Amsterdam, The Netherlands; 6 Cancer Center Amsterdam, Amsterdam, The Netherlands

## Abstract

**Background:**

Neoadjuvant therapy (NAT) for pancreatic ductal adenocarcinoma (PDAC) is increasingly being used. The aim of this study was to evaluate the association between type, duration, and sequencing of adjuvant therapy (AT) after NAT and overall survival (OS) in patients with resected PDAC.

**Methods:**

Patients receiving NAT and resection for PDAC (2010–2019) at two high-volume pancreatic surgery centers were included and stratified into groups on the basis of NAT regimen: gemcitabine-based NAT, 5-fluorouracil (5FU)-based NAT, or switched NAT regimen. The maximally selected rank statistic was used to determine the optimal NAT duration. Univariate and multivariable Cox proportional hazards models were used to assess the association between NAT regimen and OS, and between AT and OS.

**Results:**

Of 651 patients, 200 (30.7%) received gemcitabine-based NAT, 362 (56%) received 5FU-based NAT, and 89 (13.7%) switched NAT regimen. Median OS in patients receiving gemcitabine-based NAT was 19 months (95% confidence interval (CI) 17–25 months), compared with 26 months (95% CI 24–31 months) in patients receiving 5FU-based NAT (hazard ratio (HR) 0.81, 95% CI 0.66–0.99, *p* = 0.04) and 21 months (95% CI 16–26 months) in patients who switched NAT regimen (HR 0.98, 95% CI 0.73–1.29, *p* = 0.86). Optimal NAT duration was 3.6 months in the complete cohort. Receiving AT was associated with improved survival (HR 0.61, 95% CI 0.43–0.86, *p* < 0.001), but its association was reduced after a NAT duration of ≥5 months (adjuvant chemotherapy × NAT duration ≥ 5 months: HR 1.50, 95% CI 1.00–2.24, *p* = 0.048).

**Conclusions:**

Patients receiving 5FU-based NAT showed improved survival compared with patients receiving gemcitabine-based NAT before surgery for PDAC. Adjuvant therapy improved survival, particularly in patients with shorter NAT duration.

**Supplementary Information:**

The online version contains supplementary material available at 10.1245/s10434-025-17439-x.

Pancreatic ductal adenocarcinoma (PDAC) remains one of the leading causes of cancer-related death worldwide. Despite improvement in surgical techniques and perioperative management, the aggressive nature of systemic disease drives poor outcomes as reflected by a 5-year survival of around 13% across all patients.^1^ The introduction of multiagent systemic chemotherapeutics such as 5-fluorouracil (5FU)-based therapies and gemcitabine-combination regimens has led to modest improvements in survival when used in conjunction with surgery.^2-4^

Traditionally, adjuvant therapy (AT) has been the standard approach to systemic therapy, aiming to treat microscopic residual disease and prevent recurrence following pancreatic resection.^5^ Recently, an induction/neoadjuvant therapy (NAT) approach has demonstrated survival benefit for borderline resectable and locally advanced disease, and this approach is now being adopted for resectable disease.^6-8^ NAT offers potential benefits, including early control of systemic disease, improved selection of surgical candidates, and making sure that all patients receive some chemotherapy during their clinical course.^9^

Considering the increased use of NAT for PDAC, the role of AT could be debated. With prolonged exposure to preoperative chemotherapeutics, the need for and efficacy of additional postoperative chemotherapy remain unclear. Furthermore, it is unknown whether different durations of NAT affect the potential benefit of AT. Understanding this is critical in optimizing personalized treatment protocols for PDAC. There is limited evidence directly addressing the type and total duration of combined NAT and AT for PDAC, with most studies focusing on each modality alone.^10,11^ While NAT has shown survival benefits, the impact of adding or prolonging AT after NAT remains unclear and is still under investigation.

The role of adjuvant chemotherapeutics may have changed given the increased use of chemotherapeutics in the neoadjuvant setting. The aim of the study was to evaluate the association between the type, duration, and sequencing of systemic therapy and overall survival (OS) in patients with resected PDAC.

## Methods

### Study Design and Participants

This retrospective cohort study was performed at two high-volume centers for pancreatic surgery in the USA (New York University Langone Health and the Johns Hopkins Hospital). Consecutive patients who received 5FU-based or gemcitabine-based NAT in combination with pancreatic resection for histologically confirmed PDAC between 2010 and 2019 were identified from prospectively maintained databases. Patients with unknown neoadjuvant or adjuvant regimen, less than 4 weeks of NAT, R2 resections, and mortality within 90 days after resection were excluded.

This study was carried out in compliance with the ethical principles for medical research involving human subjects outlined in the Declaration of Helsinki. The institutional review boards of each participating center approved this study. The study was performed in accordance with the Strengthening the Reporting of Observational studies in Epidemiology (STROBE) guidelines.

### Objectives

The main objectives of this study were to assess overall survival (OS) after receiving gemcitabine-based or 5FU-based NAT and resection for PDAC, and to assess the impact of receiving AT after receiving different durations and types of NAT. OS was defined as the time between resection and death or last follow-up.

Patients were stratified into three groups on the basis of NAT regimen: (1) gemcitabine-based NAT, (2) 5FU-based NAT, and (3) switched NAT regimen. In general, patients were managed by a multidisciplinary team and NAT regimen was chosen following the National Comprehensive Cancer Network (NCCN) guidelines for PDAC and in a multidisciplinary setting (including surgeons, oncologists, radiation oncologists, pathologists, and radiologists).^12,13^

### Data Collection

Baseline and perioperative data were extracted from the prospectively maintained institutional databases at New York University Langone Health and the Johns Hopkins Hospital. Baseline data included age, sex, Charlson Comorbidity Index (CCI) score, American Society of Anaesthesiologists (ASA) score, resectability status according to the National Comprehensive Cancer Network (NCCN) criteria,^12^ serum carbohydrate antigen 19-9 (CA19-9) levels at diagnosis and before surgery, type and duration of neoadjuvant chemotherapy, neoadjuvant radiation, and approach and type of pancreatectomy.

Data obtained from the pathological analysis included tumor size, grade of tumor differentiation, resection margin status, microscopic perineural and lymphovascular invasion, and treatment response following the College of American Pathologists (CAP) pancreatic protocol.^14^

Postoperative data included type of adjuvant chemotherapy, vital status, and date of death or last follow-up.

The age of 65 years was chosen as the cutoff to define older age.^15^ Based on CA19-9 serum levels, patients were categorized into non-secretors (< 5 units/mL), normal (5-37 units/mL), and elevated (≥ 37 units/mL), as well as low (< 100 units/mL) and high baseline CA19-9 (≥ 100 units/mL). A microscopic clearance margin of less than 1 mm was considered positive (R1). Nodal disease stages N1 and N2 were combined into positive nodal disease.

### Statistical Analysis

Descriptive statistics were obtained. Normally distributed continuous variables were expressed as mean with standard deviation (SD) and compared using Student’s *t*-test or analysis of variance (ANOVA). Non-normally distributed continuous variables were expressed as median with interquartile range (IQR) and compared using the Mann–Whitney *U* test or Kruskal–Wallis test. Categorical values were expressed as absolute number and percentage and compared using the chi-squared test. Missing data were handled using multiple imputations with the iterative Markov chain Monte Carlo method (five imputations, ten iterations) as they were considered missing at random.^16^

Kaplan–Meier survival curves were used to estimate median OS with corresponding 95% confidence intervals (95% CI) in patients with gemcitabine-based NAT, 5FU-based NAT, and switched NAT and were compared using the log-rank test. Duration of NAT was assessed as a continuous variable for determination of the optimal cutoff point using maximally selected rank statistical (MSRS) analysis. A multivariable Cox proportional hazard analysis was used to evaluate whether NAT regimen was independently associated with OS, adjusting for potential confounders that are known at diagnosis (age ≥ 65 versus < 65 years, female versus male sex, CA19-9 level at diagnosis ≥ 100 versus < 100 U/mL, borderline resectable versus resectable and locally advanced versus resectable pancreatic cancer). In addition, a multivariable Cox proportional hazard analysis was performed to assess whether receiving adjuvant chemotherapy was associated with OS after adjusting for potential confounders known at time of administration of adjuvant chemotherapy (age ≥ 65 versus <65 years, CA19-9 level at diagnosis ≥ 100 versus < 100 U/mL, borderline resectable and locally advanced versus resectable pancreatic cancer, 5FU-based NAT and switched NAT regimen versus gemcitabine-based NAT, duration of NAT [continuous], poor/undifferentiated versus well/poor, and unknown/missing versus well/poor tumor differentiation, positive versus negative nodal disease, positive versus negative perineural invasion, R1 versus R0 resection margin, and moderate/poor versus complete/marked treatment response). To adjust for potential immortal time bias in patients receiving AT, a landmark analysis including only patients with OS longer than or equal to 4 months was performed in the multivariable analysis, including AT as a factor. To investigate whether the effect of AT on survival is modified by the duration of NAT, we included an interaction term between adjuvant chemotherapy and duration of NAT. Multicollinearity between factors was assessed using variable inflation factors.

Patients were censored at the date of death or last follow-up. A two-tailed *p*-value of < 0.05 was considered to indicate statistical significance. Statistical analysis was performed using R version 4.4.1 (Bell Laboratories, NH, USA), including the dplyr, tidyverse, mice, survival, and maxstat packages.

## Results

### Study Population and Baseline Characteristics

A total of 729 patients with NAT and resection for PDAC were identified. Of this group, 78 patients were excluded because of < 4 weeks or unknown duration of NAT, unknown NAT or AT regimen, or 90-day postoperative mortality. Therefore, 651 patients with NAT were included in the analysis (Supplementary Table 1), with a median follow-up of 42.3 months (IQR 22.9–53.6 months) in patients alive at last follow-up.

Mean age of the cohort was 65 ± 9 years, and 310 patients (47.6%) were male. A total of 172 patients (26.4%) had resectable disease, while 297 patients (45.6%) had borderline resectable disease and 182 patients (28.0%) had locally advanced disease. Median CCI was 1 (IQR 0–2), and median ASA score was 3 (IQR 3–3). Neoadjuvant radiation therapy was administered in 429 (65.9%) patients. Surgical procedure encompassed a pancreatoduodenectomy in 461 patients (70.8%).

### Neoadjuvant Treatment Groups

Of the 651 patients, 200 patients (30.7%) received gemcitabine-based NAT, 362 patients (55.6%) received 5FU-based NAT, and 89 patients (13.7%) switched NAT regimen. Baseline, perioperative, and postoperative characteristics of all three cohorts are presented in Supplementary Table 2. Groups differed significantly with regard to age, NCCN stage, CA19-9 level at diagnosis, receipt of neoadjuvant radiation therapy, CA19-9 level before surgery, surgical procedure, microscopic perineural and lymphovascular invasion, nodal disease, response to NAT, and receipt of adjuvant chemotherapy (all *p* < 0.05).

Comparisons of patients with 5FU-based NAT compared with gemcitabine-based NAT are presented in Table 1. Patients receiving 5FU-based NAT were younger, more frequently were males, had a worse NCCN stage, had a lower CA19-9 level at diagnosis, and more frequently had normalized CA19-9 levels after NAT (all *p* < 0.05). Neoadjuvant radiotherapy was administered with the same frequency (respectively 232 patients (64.1%) versus 128 patients (64.0%), *p* = 1.00) and median NAT duration was comparable (respectively 3.3 months, IQR 2.3–4.9 months versus 3.7 months, IQR 2.8–5.0 months, *p* = 0.116). AT was administered with the same frequency in both NAT groups, however, patients receiving 5FU-based NAT more frequently received 5FU-based AT compared with patients receiving gemcitabine-based NAT (*p* = 0.004).

**Table 1:** Baseline, perioperative, and postoperative characteristics of 651 patients with resection for PDAC and gemcitabine-based or 5FU-based NAT

	Gemcitabine-based NAT (*n* = 200)	5FU-based NAT (*n* = 362)	*p*-value
Age, mean (SD), years	67.6 (8.9)	62.7 (8.9)	**< 0.001**
Age ≥ 65 years, *n* (%)	128 (64.0)	155 (42.8)	**< 0.001**
Male sex, *n* (%)	82 (41.0)	187 (51.5)	**0.029**
CCI, median (IQR))	1 (0–2)	1 (0–2)	0.092
ASA score, median (IQR)	3 (3–3)	3 (3–3)	0.163
NCCN stage, *n* (%)			**0.012**
Resectable	64 (32.2)	93 (25.6)	
Borderline resectable	95 (47.2)	159 (43.8)	
Locally advanced	41 (20.6)	110 (30.6)	
CA19-9 level at diagnosis, median (IQR), U/mL	288 (82–731)	150 (51–585)	**0.001**
CA19-9 level at diagnosis, *n* (%)			**0.001**
< 100 U/mL	53 (26.7)	151 (41.6)	
≥ 100 U/mL	147 (73.3)	211 (58.4)	
Neoadjuvant radiation therapy, *n* (%)	128 (64.0)	232 (64.1)	1.000
Duration of NAT, median (IQR), months	3.3 (2.3–4.9)	3.7 (2.8–5.0)	0.089
CA19-9 level before surgery, median (IQR), U/mL	48 (22–125)	38 (18–108)	**0.017**
CA19-9 level before surgery, *n* (%)			**0.015**
Normal	60 (29.8)	146 (40.4)	
Elevated	128 (64.2)	185 (51.0)	
Non-responders	12 (6.0)	31 (8.6)	
Surgical approach, *n* (%)			0.340
Open	194 (96.9)	349 (96.5)	
Laparoscopic	2 (1.0)	4 (1.2)	
Robotic	4 (2.1)	9 (2.3)	
Surgical procedure, *n* (%)			0.158
Pancreatoduodenectomy	148 (74.0)	247 (68.2)	
Distal pancreatectomy	45 (22.5)	91 (25.1)	
Total pancreatectomy	5 (2.5)	9 (2.5)	
Other type of pancreatectomy	2 (1)	15 (4.1)	
T status, *n* (%)			0.891
T1/T2	168 (84.1)	298 (82.4)	
T3/T4	32 (15.9)	64 (17.6)	
Tumor differentiation, *n* (%)			0.226
Well/moderate	121 (60.5)	217 (59.9)	
Poor/undifferentiated	52 (26.0)	111 (30.7)	
Unknown/missing	27 (13.5)	34 (9.4)	
R1 resection margin status, *n* (%)	36 (18.0)	60 (16.6)	0.754
Microscopic perineural invasion, n (%)	136 (68.0)	222 (61.3)	0.138
Microscopic lymphovascular invasion, *n* (%)	93 (46.5)	111 (30.8)	**< 0.001**
Positive nodal disease, *n* (%)	110 (55.0)	140 (38.7)	**< 0.001**
Response to NAT, *n* (%)			**0.036**
Complete/marked	40 (20.0)	103 (28.5)	
Moderate/poor	160 (80.0)	259 (71.5)	
Adjuvant chemotherapy, *n* (%)	103 (51.5)	194 (53.6)	0.699
Type of adjuvant chemotherapy, *n* (%)			**0.001**
No adjuvant	97 (48.5)	168 (46.4)	
Gemcitabine-based	76 (38.0)	100 (27.6)	
5FU-based adjuvant	27 (13.5)	94 (26.0)	
Adjuvant radiotherapy, *n* (%)	7 (3.3)	3 (1.0)	0.624
Deceased, *n* (%)	164 (82.0)	259 (71.5)	**0.008**
Overall survival, median (95% CI)*	19 (17–25)	26 (24–31)	**0.017**

Bold indicates significant values *p* < 0.05

PDAC, pancreatic ductal adenocarcinoma; 5FU, 5-fluorouracil; NAT, neoadjuvant treatment; SD, standard deviation; CCI, Charlson Comorbidity Index; IQR, interquartile range; ASA, American Society of Anesthesiologists; NCCN, National Comprehensive Cancer Network; CA 19-9, carbohydrate antigen 19-9; NA, not applicable; CI, confidence interval

^*^Overall survival was defined as the time between resection and death or last follow-up

### Survival after Neoadjuvant and Adjuvant Treatment

**Fig. 1:**
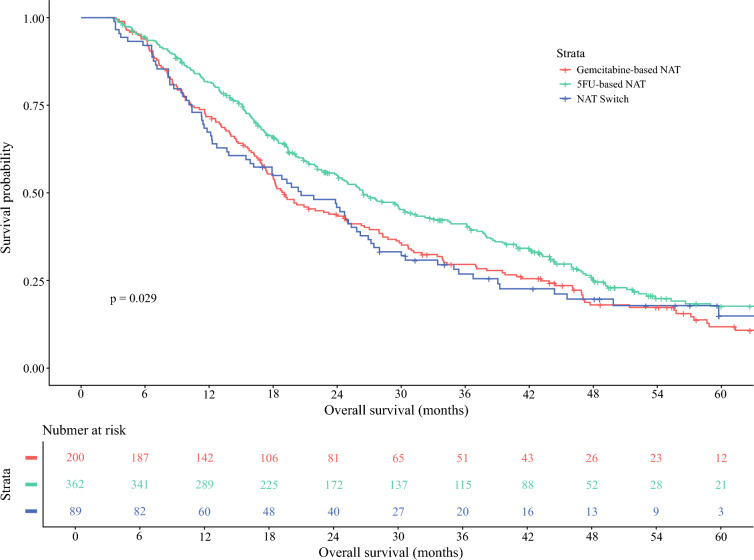
Kaplan–Meier curve comparing overall survival in patients with different NAT regimens for PDAC

Median OS was 19 months (95% CI 17–25 months) in patients receiving gemcitabine-based NAT, 26 months (95% CI 24–31 months) in patients receiving 5FU-based NAT, and 21 months (95% CI 16–26 months) in patients who switched NAT regimen (*p* = 0.029) (Fig. 1). When adjusting for potential confounders that are known before initiation of NAT, 5FU-based NAT was independently associated with improved survival compared with gemcitabine-based NAT (HR 0.81, 95% CI 0.66–0.99, *p* = 0.04), while switch of NAT type compared with gemcitabine-based NAT was not (HR 0.98, 95% CI 0.73–1.29, *p* = 0.86) (Table 2).

**Table 2:** Multivariable Cox regression analysis to identify baseline factors associated with overall survival in patients with NAT and resection for PDAC

	Overall survival	
	HR (95% CI)	*p*-value
Age (≥ 65 versus < 65 years)	1.06 (0.88–1.27)	0.528
Sex (female versus male)	0.93 (0.78–1.11)	0.421
CA19-9 level at diagnosis (≥ 100 U/mL versus < 100 U/mL)	1.34 (1.11–1.63)	**0.002**
NCCN stage		
Resectable	*ref*	*ref*
Borderline resectable	1.43 (1.14–1.79)	**0.002**
Locally advanced	1.22 (0.95–1.56)	0.128
NAT type		
Gemcitabine-based	*ref*	*ref*
5FU-based	0.81 (0.66–0.99)	**0.037**
Switched	0.98 (0.73–1.29)	0.863

Bold indicates significant values *p* < 0.05

NAT, neoadjuvant treatment; PDAC, pancreatic ductal adenocarcinoma; HR, hazard ratio; CI, confidence interval; CA19-9, carbohydrate antigen 19-9; NCCN, National Comprehensive Cancer Network; 5FU, 5-fluorouracil

When adjusting for potential confounders that are known at initiation of AT, receiving adjuvant chemotherapy (HR 0.61, 95% CI 0.43–0.86, *p* = 0.005) and NAT duration (HR 0.94, 95% CI 0.90–0.99, *p* = 0.013) were independently associated with survival. The effect of adjuvant chemotherapy on survival was not modified by duration of NAT (HR 1.03, 95% CI 0.97–1.11, *p* = 0.335) (Table 3). In addition, baseline CA19-9 cutoff > 100 (HR 1.37 95% CI 1.13–1.67, *p* = 0.002), borderline versus resectable pancreatic cancer (HR 1.31 95% CI 1.03–1.65, *p* = 0.025), poor/undifferentiated versus well/moderate tumor differentiation (HR 1.46, 95% CI 1.19–1.79, *p* < 0.001), nodal disease (HR 1.37, 95% CI 1.13–1.68, *p* = 0.002), and moderate/poor versus complete/marked treatment response (HR 1.35, 95% CI 1.06–1.73, *p* = 0.015) were associated with survival (Table 3).

**Table 3:** Multivariable Cox regression analysis to identify factors associated with overall survival in patients with NAT and resection for PDAC

	Overall survival	
	HR (95% CI)	*p*-value
Adjuvant chemotherapy (yes versus no)	0.61 (0.43–0.86)	**0.005**
Duration of NAT, months (continuous)	0.94 (0.90–0.99)	**0.013**
Adjuvant chemotherapy (yes) and duration of NAT, months (continuous)*	1.03 (0.97–1.11)	0.335
Age (≥ 65 versus < 65 years)	1.01 (0.84–1.22)	0.897
CA19-9 level at diagnosis (≥ 100 U/mL versus < 100 U/mL)	1.37 (1.13–1.67)	**0.002**
NCCN stage		
Resectable	ref	ref
Borderline resectable	1.31 (1.03–1.65)	**0.025**
Locally advanced	1.2 (0.92–1.56)	0.175
NAT type		
Gemcitabine-based	ref	ref
5FU-based	0.85 (0.69–1.05)	0.124
Switched	1.11 (0.82–1.52)	0.490
Tumor differentiation		
Well/moderate	ref	ref
Poor/undifferentiated	1.46 (1.19–1.79)	**< 0.001**
Unknown/missing	0.79 (0.56–1.12)	0.182
Nodal disease (yes versus no)	1.37 (1.13–1.68)	**0.002**
Microscopic perineural invasion (yes versus no)	1.12 (0.90–1.40)	0.295
Resection margin status (R1 versus R0)	1.16 (0.92–1.47)	0.199
Treatment response (moderate/poor versus completed/marked)	1.35 (1.06–1.73)	**0.015**

Bold indicates significant values *p* < 0.05

NAT, neoadjuvant treatment; PDAC, pancreatic ductal adenocarcinoma; HR, hazard ratio; CI, confidence interval; CA19-9, carbohydrate antigen 19-9; NCCN, National Comprehensive Cancer Network; 5FU, 5-fluorouracil

^*^ Interaction term

### Duration of NAT and Adjuvant Chemotherapy

**Fig. 2:**
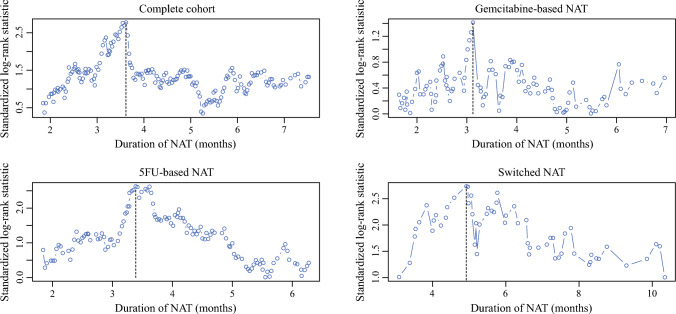
Maximally selected rank statistics for the optimal duration of NAT in different NAT groups

The maximally selected rank statistics, optimal duration of NAT of the overall cohort was 3.6 months: 3.1 months in patients receiving gemcitabine-based NAT, 3.4 months in patients receiving 5FU-based NAT, and 4.9 months in patients who switched NAT (Fig. 2).

After adjusting for potential confounders, the effect of adjuvant chemotherapy on survival was reduced after a duration of NAT of more than or equal to 5 months (adjuvant chemotherapy (yes) × duration of NAT (≥ 5 months): HR 1.50, 95% CI 1.00–2.24, *p* = 0.048) (Supplementary Table 3).

## Discussion

In this study, we evaluated the impact of different NAT regimens, duration of therapy, and subsequent adjuvant chemotherapy on survival in patients who underwent resection for PDAC. In our cohort, 5FU-based NAT was associated with improved survival compared with gemcitabine-based NAT. Patients who received at least three months of NAT had improved survival. Furthermore, the survival benefit associated with adjuvant chemotherapy was reduced after receiving more than five months of NAT. This study advances our knowledge on the importance of preoperative regimen selection and optimal treatment duration in an era of increased use of NAT, as well as the role of subsequent adjuvant chemotherapy in further improving patient outcomes.

The survival benefit of NAT with 5FU-based regimens compared with gemcitabine-based regimens remains a topic of debate.^17^ Our findings are consistent with prior studies suggesting improved survival of 5FU-based regimens over gemcitabine-based regimens in treating PDAC.^11^ Yet, the results of this study have to be interpreted while considering differences between these patient cohorts. Patients receiving 5FU-based NAT had better baseline characteristics (i.e., were younger and had lower CA19-9 level at diagnosis) compared with patients receiving gemcitabine-based NAT, even though they had more advanced disease stage when considering NCCN stage. Besides this, 5FU-based NAT was associated with higher rates of treatment response compared with gemcitabine-based NAT looking at the number of normalized preoperative CA19-9 levels as well as less perineural and lymphovascular invasion, nodal disease, and more complete or near complete pathological treatment response. Notably, switching NAT regimens did not offer any survival advantage compared with patients receiving gemcitabine-based NAT in our cohort. This may suggest that 5FU-based NAT should be initiated for all patients who are eligible to receive NAT, even though its potential for being more toxic and higher risk of need for switch of therapy due to poor tolerance. However, conclusions cannot be drawn on the basis of this retrospective analysis, and these findings should be further explored in prospective randomized trials to confirm their validity and implications.

In contrasts to our results, recent studies have not showed improved survival after 5FU-based NAT over gemcitabine-based NAT.^18,19^ One potential reason for this discrepancy is the high frequency of radiation therapy (i.e., stereotactic body radiotherapy) in both groups of the current study (64.1% and 64.0%, respectively). Previous studies have not included such high numbers of radiation therapy, or radiation therapy was given solely in the gemcitabine-based NAT group.^18,20^ Combing radiation therapy with chemotherapy can improve local tumor control through multiple mechanisms including sensitizing cancer cells to radiation-induced DNA damage, making them more susceptible to the cytotoxic effects of chemotherapy. It has also been suggested that fluoropyrimidines are known to act as radiosensitizers, potentially enhancing the efficacy of concurrent radiation therapy more than gemcitabine-based regimens.^21^ This synergistic effect of radiation therapy combined with chemotherapy could have contributed to the better survival mainly in the setting of 5FU-based NAT. Even though this may highlight the role of multimodal therapy in improving treatment response, the role of radiation therapy in the neoadjuvant and adjuvant setting of PDAC treatment, in combination with different chemotherapeutics including 5FU-based regimens, has yet to be demonstrated as survival benefits have not yet been shown.^22,23^

Beyond NAT regimen choice, several factors were associated with overall survival, emphasizing the complex interplay between tumor biology and treatment outcomes. In this regard, a higher baseline CA19-9 level (≥ 100 U/mL) was prognostic for poorer survival in our study. Even though this biomarker has previously been shown to be associated with more advanced disease, several studies have suggested that baseline CA19-9 levels loses its prognostic value during NAT, and the use of CA19-9 dynamics or static preoperative CA19-9 levels is suggested.^24,25^ In our study, it could be seen that patients receiving 5FU-based NAT more frequently had normalized CA19-9 levels before surgery compared with patients receiving gemcitabine-based NAT. CA19-9 dynamics can thus potentially guide as an early indication of treatment response that has been shown to be associated with improved survival.^26^ In this study, it was seen that response to NAT was associated with improved survival, and patients receiving 5FU-based NAT were more likely to have complete or near complete treatment response.

In our study, patients with borderline resectable tumors had decreased survival compared with those with resectable disease, while locally advanced tumors were not associated with decreased survival. Previous studies have suggested that the prognostic significance of preoperative resectability status drops out with comparable survival for different NCCN stages.^27,28^ These findings highlight the need for better prognostication to assess and predict tumor response during and after neoadjuvant therapy.^13^

Even though duration of NAT was significantly associated with improved survival on multivariable analysis, our maximally selected rank statistics showed an optimal NAT duration of approximately 3.6 months in the current cohort, with variations based on NAT regimen. We hypothesized that, from this cutoff onwards, the NAT has caused sufficient systemic disease control without causing unnecessary delay in surgery and thus local disease control. Patients might still be eligible to receive AT after surgery, completing all necessary months of chemotherapy and further treating microscopic residual disease. Still, we do not advocate giving 3.6 months of NAT only, as some patients might not be eligible to complete systemic treatment in the form of AT after surgery, as well as the fact that these numbers are based on retrospective data in a selected cohort. After all, our results suggest that the addition of AT was associated with significantly better survival, which is in line with previous research.^29^ This study thereby underscores the importance of AT to achieve long-term disease control and prevent (early) disease recurrence, especially for patients who may have undergone suboptimal NAT duration. Previous studies and consensus guidelines have suggested that a total duration of approximately 6 months of systemic therapy may be optimal for patients with PDAC. This approach is extrapolated from adjuvant chemotherapy trials, such as the ESPAC-4 and PRODIGE 24 trials.^10,11^ Although clinicians often aim for a similar total duration of combined NAT and AT, (prospective) studies evaluating a combined NAT and AT duration are lacking.

There are several limitations to this study. First, the retrospective nature of the analysis limits the ability to prove causality. Even after adjusting for potential confounders, residual bias may exist. Second, despite though being a bi-institutional study, the cohort encompassed patients from the USA only, potentially limiting the generalizability of our findings. Third, survival differences in patients receiving adjuvant chemotherapy or not may be affected by immortal time bias. To address this, we performed a landmark analysis including only patients with OS longer than or equal to 4 months. Given current guidelines recommending the initiation of adjuvant chemotherapy within 12 weeks after surgery, this approach mitigated immortal time bias. However, residual bias cannot be excluded. Fourth, accounting for the number of NAT cycles and potential dose reductions could have provided a more accurate assessment of the chemotherapy intensity compared with NAT duration in months only. However, this was beyond the scope of our study. Finally, even though this study included a relatively large sample size, the number of patients was limited and larger global efforts are required to further dissect the findings in different subgroups such as in the context of radiotherapy or based on NCCN stage, NAT regimen, and AT regimen.

In conclusion, patients receiving 5FU-based NAT in our study had improved overall survival compared with patients receiving gemcitabine-based NAT before surgery for PDAC. Moreover, patients receiving adjuvant chemotherapy had improved survival after PDAC resection, particularly these with shorter duration of NAT. These results suggest that a personalized approach to selection of adjuvant therapy that factors in the type and duration of neoadjuvant therapy could help optimize management in patients with resected PDAC. Large cohort studies are required in the future to validate these findings and improve our current understanding of the nuances of systemic therapy utilization in these patients.

## Supplementary Information

Below is the link to the electronic supplementary material.

Supplementary file1 (DOCX 32 KB)
