# Supplementary material for: Association Between Adjuvant Therapy and Survival in Resected Pancreatic Ductal Adenocarcinoma After Different Types and Durations of Neoadjuvant Therapy
Source: Ann Surg Oncol. 2025 May 29;32(9):6550–8. doi: 10.1245/s10434-025-17439-x (PMC12317898; doi:10.1245/s10434-025-17439-x)
Supplement: Supplementary file 1 — Supplementary file1 (DOCX 32 KB) [file 10434_2025_17439_MOESM1_ESM.docx]

**Association Between Adjuvant Therapy and Survival in Resected Pancreatic Ductal Adenocarcinoma After Different Types and Durations of Neoadjuvant Therapy**

**SUPPLEMENTS**

| ***Supplemental table 1.*** *Baseline, perioperative and postoperative characteristics and missing data of 651 patients who received NAT and resection for PDAC* | | | |
| --- | --- | --- | --- |
|  | **Before imputation** | **Missing values, n (%)** | **After imputation** |
| Age, mean (SD) | 64.5 (9) | - | 64.5 (9) |
| Age ≥65, n (%) | 328 (50.4) | - | 328 (50.4) |
| Male sex, n (%) | 286 (48.5) | 61 (9.4) | 310 (47.6) |
| CCI, median (IQR) | 1 (0-2) | 170 (26.1) | 1 (0-2) |
| ASA score, median (IQR) | 3 (3-3) | 37 (5.7) | 3 (3-3) |
| NCCN status, n (%) |  | 195 (30.0) |  |
| Resectable | 118 (25.9) |  | 172 (26.4) |
| Borderline resectable | 210 (46.1) |  | 297 (45.6) |
| Locally advanced | 128 (28.1) |  | 182 (28.0) |
| CA19-9 level at diagnosis, median (IQR), U/mL | 217 (62-700) | 65 (10.0) | 217 (60-700) |
| CA19-9 level at diagnosis, n (%) |  | 65 (10.0) |  |
| <100 U/mL | 207 (35.3) |  | 231 (35.5) |
| ≥100 U/mL | 379 (64.7) |  | 420 (64.5) |
| NAT type, n (%) |  | - | NA |
| Gemcitabine-based | 200 (30.7) |  |  |
| 5FU-based | 362 (55.6) |  |  |
| Switched | 89 (13.7) |  |  |
| Type of NAT switch, n (%) |  | - | NA |
| Gemcitabine-based to 5FU-based | 15 (16.9) |  |  |
| 5FU-based to gemcitabine-based | 74 (83.1) |  |  |
| Neoadjuvant radiation therapy, n (%) | 429 (65.9) | - | 429 (65.9) |
| Duration of NAT, median (IQR), months | 3.78 (2.64, 5.31) | - | NA |
| CA19-9 level before surgery, median (IQR), U/mL | 43 (20-113) | 100 (15.4) | 42 (20-114) |
| CA19-9 level before surgery, n (%) |  | 100 (15.4) |  |
| Normal | 212 (38.5) |  | 241 (36.9) |
| Elevated | 310 (56.3) |  | 363 (55.8) |
| Non-responders | 29 (5.3) |  | 47 (7.3) |
| Surgical approach, n (%) |  | 105 (16.1) |  |
| Open | 534 (97.8) |  | 630 (96.7) |
| Laparoscopic | 3 (0.5) |  | 6 (1.0) |
| Robotic | 9 (1.6) |  | 15 (2.2) |
| Surgical procedure, n (%) |  | - |  |
| Pancreatoduodenectomy | 461 (70.8) |  | 461 (70.8) |
| Distal pancreatectomy | 154 (23.7) |  | 154 (23.7) |
| Total pancreatectomy | 15 (2.3) |  | 15 (2.3) |
| Other type of pancreatectomy | 21 (3.2) |  | 21 (3.2) |
| Tumor size, mean (SD) | 25 (16) | - | 25 (16) |
| T-status, n (%) |  | 42 (6.5) |  |
| T1/T2 | 502 (82.4) |  | 535 (82.2) |
| T3/T4 | 107 (17.6) |  | 116 (17.8) |
| Tumor differentiation, n (%) |  | - |  |
| Well/moderate | 394 (60.5) |  | 394 (60.5) |
| Poor/undifferentiated | 188 (28.9) |  | 188 (28.9) |
| Unknown/missing | 69 (10.6) |  | 69 (10.6) |
| R1 resection margin status, n (%) | 115 (17.7) | - | 115 (17.7) |
| Microscopic perineural invasion, n (%) | 413 (63.4) | - | 413 (63.4) |
| Microscopic Lymphovascular invasion, n (%) | 220 (36.0) | 40 (6.1) | 236 (36.2) |
| Positive nodal disease, n (%) | 286 (43.9) | - | 286 (43.9) |
| Response to NAT, n (%) |  | - | NA |
| Complete/marked | 165 (25.3) |  | 165 (25.3) |
| Moderate/poor | 486 (74.7) |  | 486 (74.7) |
| Adjuvant chemotherapy, n (%) | 341 (52.4) | - | NA |
| Type of adjuvant chemotherapy, n (%) |  | - | NA |
| No adjuvant | 310 (47.6) |  |  |
| Gemcitabine-based | 215 (33.0) |  |  |
| 5FU-based adjuvant | 126 (19.4) |  |  |
| Adjuvant radiotherapy, n (%) | 6 (1.0) | 54 (8.3) | 10 (1.6) |
| Deceased, n (%) | 495 (76.0) | - | NA |
| *NAT, neoadjuvant treatment; SD, standard deviation; CCI, Charlson Comborbidity Index; IQR, interquartile range; ASA, American Society of Anesthesiologists; NCCN, National Comprehensive Cancer Network; CA19-9, carbohydrate antigen 19-9; NA, not applicable; 5FU, 5-fluorouracil.* | | | |

| ***Supplemental Table 2.*** *Baseline, perioperative and postoperative characteristics of 651 patients with resection for PDAC stratified by NAT type* | | | | |
| --- | --- | --- | --- | --- |
|  | **Gemcitabine-based NAT**  **(n = 200)** | **5FU-based NAT**  **(n = 362)** | **Switched NAT**  **(n = 89)** | **p-value** |
| Age, mean (SD), years | 67.6 (8.9) | 62.7 (8.9) | 64.3 (8.1) | <0.001 |
| Age ≥65 years, n (%) | 128 (64) | 155 (42.8) | 45 (50.6) | <0.001 |
| Male sex, n (%) | 82 (41) | 187 (51.5) | 41 (46.5) | 0.068 |
| CCI, median (IQR)) | 1 (0-2) | 1 (0-2) | 0 (0-1) | 0.060 |
| ASA score, median (IQR) | 3 (3-3) | 3 (3-3) | 3 (3-3) | 0.349 |
| NCCN stage, n (%) |  |  |  | 0.006 |
| Resectable | 64 (32.2) | 93 (25.6) | 15 (16.4) |  |
| Borderline resectable | 95 (47.2) | 159 (43.8) | 44 (49.4) |  |
| Locally advanced | 41 (20.6) | 110 (30.6) | 30 (34.2) |  |
| CA19-9 level at diagnosis, median (IQR), U/mL | 288 (82-731) | 150 (51-585) | 380 (68-1394) | <0.001 |
| CA19-9 level at diagnosis, n (%) |  |  |  | 0.001 |
| <100 U/mL | 53 (26.7) | 151 (41.6) | 27 (30.1) |  |
| ≥100 U/mL | 147 (73.3) | 211 (58.4) | 62 (69.9) |  |
| Neoadjuvant radiation therapy, n (%) | 128 (64) | 232 (64.1) | 69 (77.5) | 0.045 |
| Duration of NAT, median (IQR), months | 3.3 (2.3-4.9) | 3.7 (2.8-5.0) | 5.7 (4.4-7.9) | <0.001 |
| CA19-9 level before surgery, median (IQR), U/mL | 48 (22-125) | 38 (18-108) | 46 (21-136) | 0.053 |
| CA19-9 level before surgery, n (%) |  |  |  | 0.013 |
| Normal | 60 (29.8) | 146 (40.4) | 35 (38.7) |  |
| Elevated | 128 (64.2) | 185 (51) | 50 (56.4) |  |
| Non-responders | 12 (6) | 31 (8.6) | 4 (4.9) |  |
| Surgical approach, n (%) |  |  |  | 0.513 |
| Open | 194 (96.9) | 349.2 (96.5) | 87 (97.5) |  |
| Laparoscopic | 2 (1) | 4.4 (1.2) | - |  |
| Robotic | 4 (2.1) | 8.4 (2.3) | 2 (2.2) |  |
| Surgical procedure, n (%) |  |  |  | 0.345 |
| Pancreatoduodenectomy | 148 (74) | 247 (68.2) | 66 (74.2) |  |
| Distal pancreatectomy | 45 (22.5) | 91 (25.1) | 18 (20.2) |  |
| Total pancreatectomy | 5 (2.5) | 9 (2.5) | 1 (1.1) |  |
| Other type of pancreatectomy | 2 (1) | 15 (4.1) | 4 (4.5) |  |
| Tumor size, mean (SD), mm | 26 (16) | 25 (16) | 27 (18) | 0.374 |
| T-status, n (%) |  |  |  | 0.443 |
| T1/T2 | 168 (84.1) | 298 (82.4) | 69 (77.1) |  |
| T3/T4 | 32 (15.9) | 64 (17.6) | 20 (22.9) |  |
| Tumor differentiation, n (%) |  |  |  | 0.493 |
| Well/moderate | 121 (60.5) | 217 (59.9) | 56 (62.9) |  |
| Poor/undifferentiated | 52 (26) | 111 (30.7) | 25 (28.1) |  |
| Unknown/missing | 27 (13.5) | 34 (9.4) | 8 (9) |  |
| R1 resection margin status, n (%) | 36 (18) | 60 (16.6) | 19 (21.3) | 0.565 |
| Microscopic perineural invasion, n (%) | 136 (68) | 222 (61.3) | 55 (61.8) | 0.273 |
| Microscopic lymphovascular invasion, n (%) | 93 (46.5) | 111 (30.8) | 31 (35.3) | 0.001 |
| Positive nodal disease, n (%) | 110 (55) | 140 (38.7) | 36 (40.4) | 0.001 |
| Response to NAT, n (%) |  |  |  | 0.087 |
| Complete/marked | 40 (20) | 103 (28.5) | 22 (24.7) |  |
| Moderate/poor | 160 (80) | 259 (71.5) | 67 (75.3) |  |
| Adjuvant chemotherapy, n (%) | 103 (51.5) | 194 (53.6) | 44 (49.4) | 0.747 |
| Type of adjuvant chemotherapy, n (%) |  |  |  | <0.001 |
| No adjuvant | 97 (48.5) | 168 (46.4) | 45 (50.6) |  |
| Gemcitabine-based | 76 (38) | 100 (27.6) | 39 (43.8) |  |
| 5FU-based adjuvant | 27 (13.5) | 94 (26) | 5 (5.6) |  |
| Adjuvant radiotherapy, n (%) | 7 (3.3) | 3 (1) | - | 0.369 |
| Deceased, n (%) | 164 (82) | 259 (71.5) | 72 (80.9) | 0.011 |
| Overall survival, median (95%CI)* | 19 (17-25) | 26 (24-31) | 21 (16-26) | 0.029 |
| *PDAC, pancreatic ductal adenocarcinoma; NAT, neoadjuvant treatment; 5FU, 5-fluorouracil; SD, standard deviation; CCI, Charlson Comborbidity Index; IQR, interquartile range; ASA, American Society of Anesthesiologists; NCCN, National Comprehensive Cancer Network; CA, 19-9, carbohydrate antigen 19-9; NA, not applicable; 95%CI, 95% confidence interval.*  **Overall survival was defined as the time between resection and death or last follow-up.* | | | | |

| ***Supplemental table 3****. Multivariable Cox regression analyses to identify factors associated with overall survival in patients with resected PDAC after different NAT durations* | | |
| --- | --- | --- |
|  | **Overall survival** | |
|  | **HR (95%CI)** | **p-value** |
| 2 months duration of NAT*^#^* |  |  |
| Adjuvant chemotherapy (yes vs no) | 0.64 (0.38-1.07) | 0.088 |
| Duration of NAT (≥2 months vs <2 months) | 0.83 (0.55-1.26) | 0.388 |
| Adjuvant chemotherapy (yes) and duration of NAT (≥2 months)* | 1.16 (0.67-2.00) | 0.596 |
|  |  |  |
| 3 months duration of NAT *^#^* |  |  |
| Adjuvant chemotherapy (yes vs no) | 0.58 (0.42-0.81) | **0.001** |
| Duration of NAT (≥3 months vs <3 months) | 0.70 (0.52-0.95) | **0.023** |
| Adjuvant chemotherapy (yes) and duration of NAT (≥3 months)* | 1.36 (0.92-2.02) | 0.121 |
|  |  |  |
| 4 months duration of NAT *^#^* |  |  |
| Adjuvant chemotherapy (yes vs no) | 0.66 (0.51-0.85) | **0.002** |
| Duration of NAT (≥4 months vs <4 months) | 0.81 (0.61-1.07) | 0.133 |
| Adjuvant chemotherapy (yes) and duration of NAT (≥4 months)* | 1.18 (0.82-1.70) | 0.384 |
|  |  |  |
| 5 months duration of NAT *^#^* |  |  |
| Adjuvant chemotherapy (yes vs no) | 0.62 (0.49-0.77) | **<0.001** |
| Duration of NAT (≥5 months vs <5 months) | 0.64 (0.48-0.86) | **0.003** |
| Adjuvant chemotherapy (yes) and duration of NAT (≥5 months)* | 1.50 (1.00-2.24) | **0.048** |
| *NAT, neoadjuvant treatment; PDAC, pancreatic ductal adenocarcinoma; HR, hazard ratio; CI, confidence interval;*  *CA19-9, carbohydrate antigen 19-9; NCCN, National Comprehensive Cancer Network; 5FU, 5-fluorouracil.*  *^#^ Other factors included in the multivariable analysis were:*  *age ≥65 vs <65 years, CA19-9 level at diagnosis ≥100 vs <100 U/mL, borderline resectable and locally advanced vs resectable pancreatic cancer, 5FU-based NAT and switched NAT regimen vs gemcitabine-based NAT, duration of NAT [continuous], poor/undifferentiated vs well/poor, and unknown/missing vs well/poor tumor differentiation, positive vs negative nodal disease, positive vs negative perineural invasion, R1 vs R0 resection margin, and moderate/poor vs complete/marked treatment response.*  ** Interaction term* | | |
